# Targeting MET and EGFR crosstalk signaling in triple-negative breast cancers

**DOI:** 10.18632/oncotarget.12065

**Published:** 2016-09-16

**Authors:** Erik S. Linklater, Elizabeth A. Tovar, Curt J. Essenburg, Lisa Turner, Zachary Madaj, Mary E. Winn, Marianne K. Melnik, Hasan Korkaya, Christiane R. Maroun, James G. Christensen, Matthew R. Steensma, Julie L. Boerner, Carrie R. Graveel

**Affiliations:** ^1^ Center for Cancer and Cell Biology, Van Andel Research Institute, Grand Rapids, Michigan, USA; ^2^ Pathology and Biorepository Core, Van Andel Research Institute, Grand Rapids, Michigan, USA; ^3^ Bioinformatics and Biostatistics Core, Van Andel Research Institute, Grand Rapids, Michigan, USA; ^4^ Spectrum Health Cancer Center, Spectrum Health System, Grand Rapids, Michigan, USA; ^5^ Grand Rapids Medical Education Partners, General Surgery Residency Program, Grand Rapids, Michigan, USA; ^6^ Department of Surgery, Michigan State University College of Human Medicine, Grand Rapids, Michigan, USA; ^7^ Molecular Oncology and Biomarkers Program, Augusta University, Augusta, Georgia, USA; ^8^ Mirati Therapeutics, San Diego, California, USA; ^9^ Biobanking and Correlative Sciences Core, Karmanos Cancer Institute, Detroit, Michigan, USA; ^10^ Current address: Vertex Pharmaceuticals (Canada) Inc., Laval, Quebec, Canada

**Keywords:** triple-negative breast cancer, receptor tyrosine kinase, MET, EGFR, tyrosine kinase inhibitors

## Abstract

There is a vital need for improved therapeutic strategies that are effective in both primary and metastatic triple-negative breast cancer (TNBC). Current treatment options for TNBC patients are restricted to chemotherapy; however tyrosine kinases are promising druggable targets due to their high expression in multiple TNBC subtypes. Since coexpression of receptor tyrosine kinases (RTKs) can promote signaling crosstalk and cell survival in the presence of kinase inhibitors, it is likely that multiple RTKs will need to be inhibited to enhance therapeutic benefit and prevent resistance. The MET and EGFR receptors are actionable targets due to their high expression in TNBC; however crosstalk between MET and EGFR has been implicated in therapeutic resistance to single agent use of MET or EGFR inhibitors in several cancer types. Therefore it is likely that dual inhibition of MET and EGFR is required to prevent crosstalk signaling and acquired resistance. In this study, we evaluated the heterogeneity of MET and EGFR expression and activation in primary and metastatic TNBC tumorgrafts and determined the efficacy of MET (MGCD265 or crizotinib) and/or EGFR (erlotinib) inhibition against TNBC progression. Here we demonstrate that combined MET and EGFR inhibition with either MGCD265 and erlotinib treatment or crizotinib and erlotinib treatment were highly effective at abrogating tumor growth and significantly decreased the variability in treatment response compared to monotherapy. These results advance our understanding of the RTK signaling architecture in TNBC and demonstrate that combined MET and EGFR inhibition may be a promising therapeutic strategy for TNBC patients.

## INTRODUCTION

Triple-negative breast cancer (TNBC) accounts for 15–20% of breast cancers and is associated with advanced stage at diagnosis and poorer outcome compared to other breast cancer subtypes [1]. TNBC is defined by the lack of expression of estrogen receptor (ER), progesterone receptor (PR), and HER2. Characteristic clinical features of TNBC include a peak risk of recurrence within the first 3 years, a peak of cancer-related death in the first 5 years, and a weak relationship between the tumor size and lymph node metastasis [2]. At the molecular level, TNBC has significant overlap with the basal-like subtype with approximately 80% of TNBCs being classified as basal-like [1]. Comprehensive gene expression analyses have revealed that TNBC is a highly heterogeneous disease that is composed of 4–6 molecular subtypes [3, 4]. This molecular heterogeneity increases the difficulty of developing targeted therapeutics that will be effective in the majority of TNBC patients.

Chemotherapy is currently the only systemic therapy available for TNBC patients, yet tyrosine kinases are potential actionable targets due to their high expression in multiple TNBC subtypes. The success of trastuzumab in HER2^+^ breast cancer underscores the promise of targeting tyrosine kinases. In spite of this promising start, tyrosine kinase inhibitors (TKIs) have had limited success in the clinic. Both innate and acquired resistance are significant clinical issues for TKIs, in part due to compensation signaling through alternative RTK pathways. Functional redundancies of vital signaling networks are often provided by activation of alternate receptor tyrosine kinases (RTKs) that maintain the tumorigenic growth in the presence of TKIs [5–7]. This signaling compensation can result from ligand activation, activating mutations, or overexpression of other RTKs that provide the cell with essential survival signals. Therefore, to successfully treat TNBC it will likely be necessary to inhibit multiple RTKs or critical signaling nodes downstream of oncogenic RTK pathways.

RTKs, in particular MET and EGFR, are promising therapeutic targets for TNBC due to their high expression in multiple molecular TNBC subtypes [3]. We and others have demonstrated that MET is highly expressed in TNBC [8–10] and its expression correlates with poor prognosis [11–15]. EGFR expression is elevated in up to 72% of basal and triple-negative breast cancers and correlates with poor prognosis in TNBC patients [16–19]. Both MET and EGFR are successful therapeutic targets in other cancers, yet have not been thoroughly tested in TNBC. Crosstalk between EGFR and MET has been implicated in therapeutic resistance to EGFR inhibitors in colon and lung cancers [20–24]. In TNBC, EGFR remains phosphorylated in the presence of EGFR inhibitors and persistent EGFR phosphorylation correlates with TKI resistance [25–29]. This resistance to EGFR inhibition may be mediated through MET-EGFR crosstalk. Therefore it is essential that we have a thorough understanding of the landscape of RTK expression in TNBC in order to develop effective treatment strategies.

Here we evaluated the efficacy of MET and/or EGFR inhibition in TNBC. In order to interrogate RTK signaling in TNBC progression and treatment, we developed and characterized patient-derived tumorgraft models of TNBC. These TNBC tumorgrafts recapitulate the genetic and molecular heterogeneity of the primary tumor [30]. In this study, we evaluated the heterogeneity of MET and EGFR expression in primary and metastatic TNBC tumorgrafts and evaluated the efficacy of MET and EGFR inhibition against tumor growth. Even though monotherapy with MET or EGFR inhibitors was effective against TNBC growth, combination of MET and EGFR inhibitors was significantly more effective. These results advance our understanding of the RTK signaling architecture in TNBC and indicate potential therapeutic strategies for TNBC patients.

## RESULTS

### MET and EGFR are highly expressed in human TNBC

**Figure 1:**
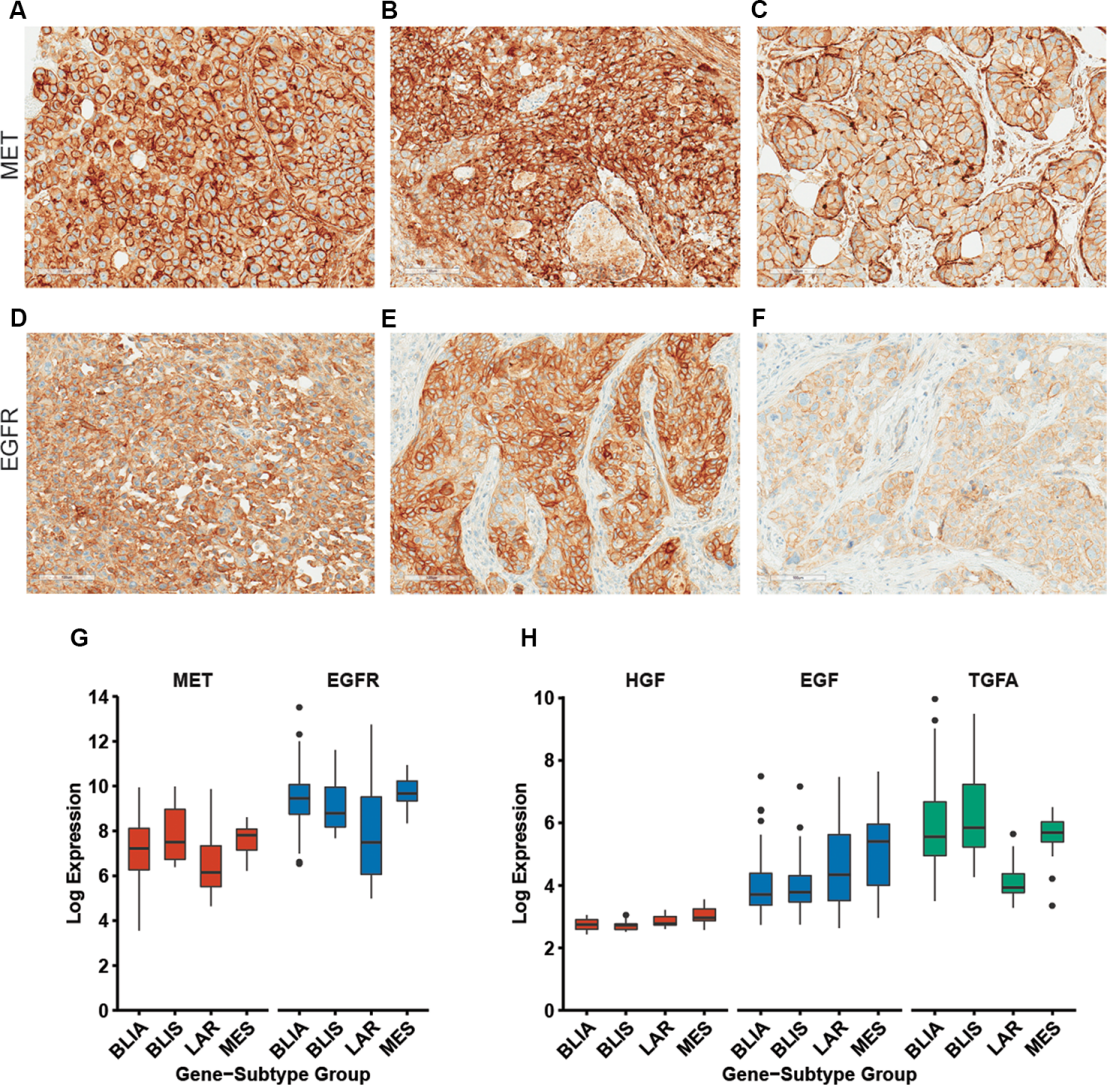
MET and EGFR are highly expressed in human TNBC subtypes (**A**–**C**) Moderate to high MET expression was observed in primary TNBC tumors by immunostaining. Distinct patterns of membrane and cytoplasmic MET expression were observed in TNBC tumors. (**D**–**F**) Immunostaining of EGFR expression revealed moderate to high membrane expression in 30% of the TNBC cases evaluated. (**G**) Gene expression analysis of MET and EGFR expression in TNBC subtypes, including the basal-like immune-activated (BLIA), basal-like immunosuppressed (BLIS), luminal androgen receptor (LAR), and mesenchymal (MES). (**H**) Gene expression analysis of the MET and EGFR ligands, hepatocyte growth factor (*HGF*) and EGFR ligands epidermal growth factor (*EGF*) and transforming growth factor α (*TGFA*) in TNBC subtypes. Data was extracted for specific Affymetrix probes from the public data set GSE2163 [4].

We and others have independently demonstrated that MET and EGFR are highly expressed in TNBC and correlate with poor patient prognosis [8–10] [17, 31, 32]. In this study, we evaluated expression patterns of both MET and EGFR in 13 TNBC cases. Overall we observed moderate to high MET expression in all of the TNBC cases; however we noticed variable patterns of MET localization. Strong membrane staining was commonly observed with varying levels of cytoplasmic expression (Figure 1A–1C). In several cases, we observed clusters of cells with robust MET staining at the periphery (Figure 1C). These variable patterns of membrane and cytoplasmic MET expression have been observed in other breast cancer studies [11, 13, 15], yet the consequences of membrane vs. cytoplasmic expression on MET signaling and clinical outcome is unclear. Previous studies have used MET antibodies that recognize the cytoplasmic domain of MET whereas the MET4 antibody used in our studies (Figure 1) recognizes the extracellular region of human MET (25 – 567). Consequently, our data indicates that full length MET is also present in the cytoplasm and is not just a cytoplasmic fragment. We also observed moderate to high EGFR expression in 30% of the TNBC cases evaluated. EGFR expression was detected primarily at the membrane using an EGFR antibody that recognizes the extracellular region (amino acids 30–198) of EGFR (Figure 1D–1F). We also detected MET staining in stromal cells within several TNBC cases. This observation is supported by a recent study in which MET activity and expression was analyzed in 18 different cancer types using quantitative digital imaging [33]. The authors observed MET staining in stromal cells within several cancers and demonstrated that the MET4 antibody correlated more strongly with active MET (phosphorylated MET) than other MET antibodies.

At the molecular level, TNBC is highly heterogeneous and this molecular heterogeneity likely underlies the variable treatment response that is observed in TNBC patients. To evaluate how extensive MET and EGFR signaling is across the TNBC molecular subtypes, we analyzed gene expression profiles of MET and EGFR in the four molecular TNBC subtypes defined by Burstein et al. [4]. The results revealed that MET and EGFR are expressed in each of the TNBC subtypes consisting of the basal-like immune-activated (BLIA), basal-like immunosuppressed (BLIS), luminal androgen receptor (LAR), and mesenchymal (MES) (Figure 1G). We also examined expression of the MET ligand hepatocyte growth factor (*HGF*) and EGFR ligands epidermal growth factor (*EGF*) and transforming growth factor α (*TGFA*) (Figure 1H). *HGF* and *EGF* were most highly expressed in the MES subtype. These findings indicate that MET and EGFR may be therapeutic targets across the diverse molecular subtypes that are present in TNBC patients.

### Patient-derived TNBC tumorgrafts recapitulate kinase diversity and have higher MET and EGFR expression

**Figure 2:**
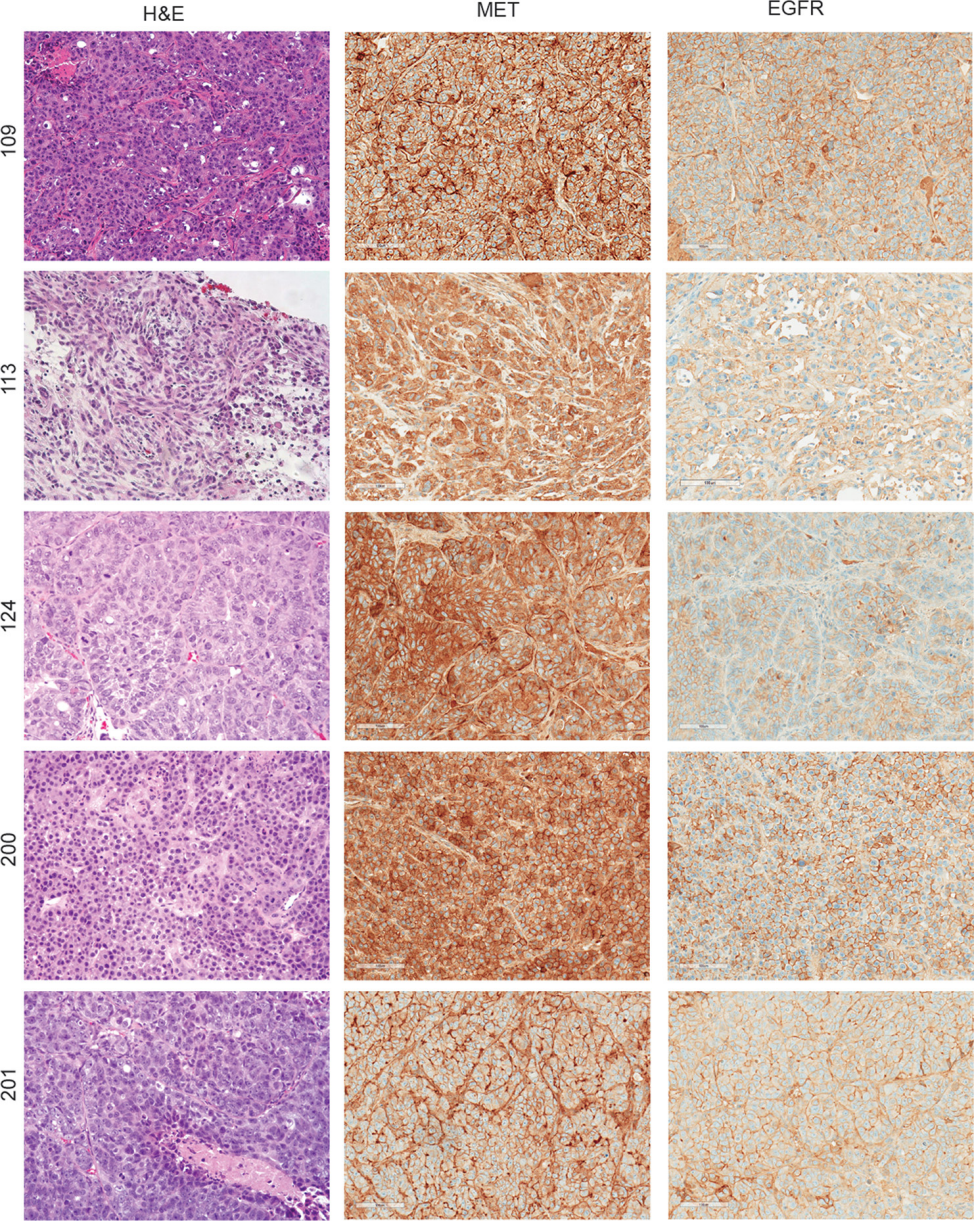
Diversity of MET and EGFR expression in patient-derived TNBC tumorgrafts Expression of MET and EGFR was determined by immunostaining in five PDX lines. PDX lines 109, 113, and 124 were established from primary TNBC tumors and the 200 and 201 lines were established from pleural effusions. Left column, hematoxylin and eosin staining; middle column, MET immunostaining; and right column, EGFR immunostaining.

We developed and characterized five patient-derived tumorgraft models from TNBC tumors that displayed significant histological diversity (Figure 2). PDX lines 109, 113, and 124 were established from primary TNBC tumors; whereas the 200 (also known as MC1) and 201 lines were established from pleural effusions [34]. We observed that the original pathological features were still present after several passages. For instance, TNBCs described as ductal adenocarcinomas (109 and 124) and a metaplastic carcinoma with spindle cell features (113) maintained these characteristics in the mouse xenografts. Distinct MET and EGFR expression patterns were observed in these TNBC tumorgraft lines (Figures 2 and Supplementary Table S1). For instance, PDX lines 113 and 201 had moderate MET expression compared to PDX lines 109, 124, and 200 which expressed high levels of MET. EGFR expression was highest in lines 109 and 200, was moderately expressed in 113 and 201, and weakly expressed in 124. This diversity in MET and EGFR expression allowed us to evaluate how variable levels of MET and EGFR expression affect downstream signaling, response to TKI treatment strategies, and the development of resistance mechanisms.

**Figure 3:**
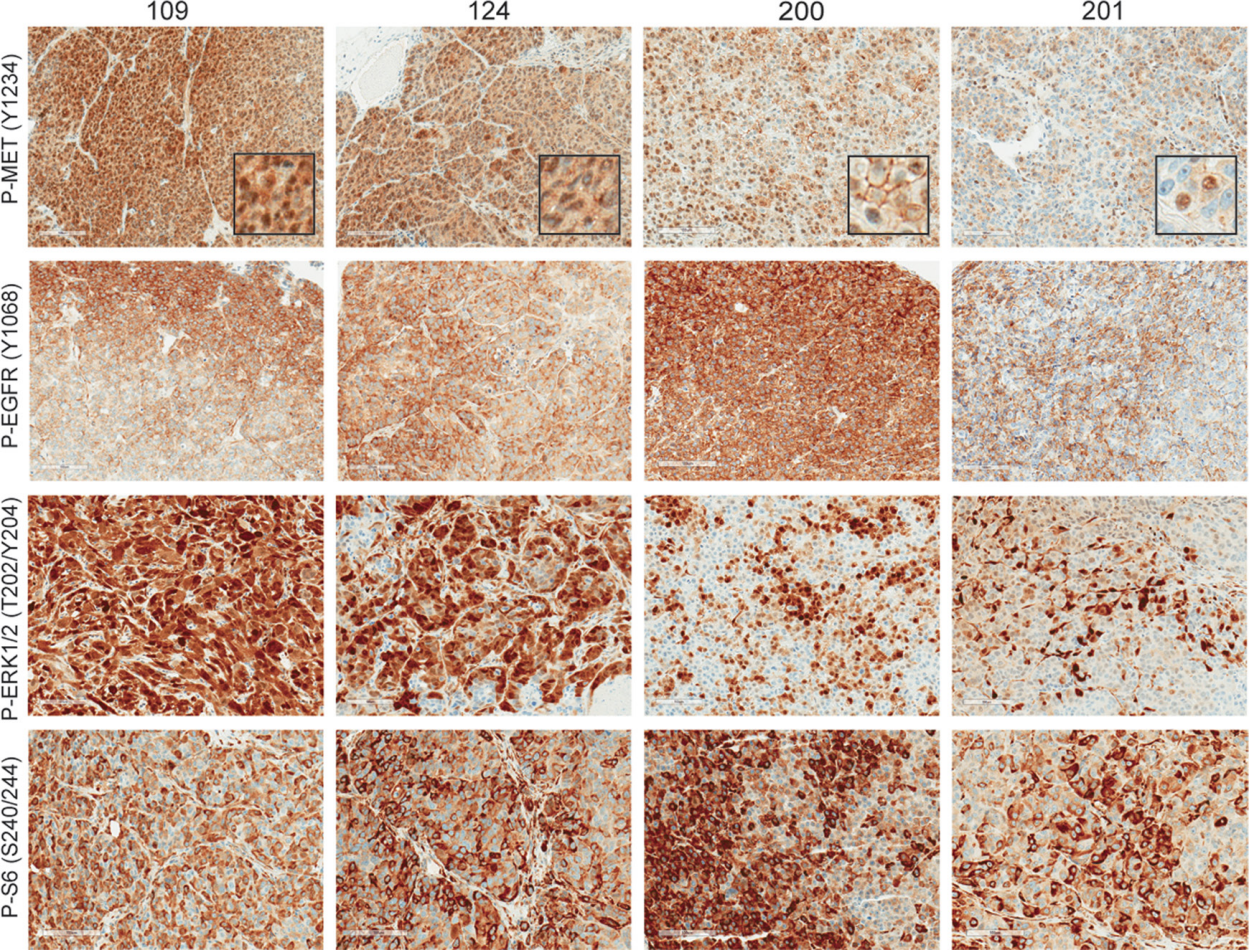
MET and EGFR signaling is highly activated in TNBC MET and EGFR activation was determined by immunostaining of P-MET (Y1234/1235), P-EGFR (Y1068), P-ERK1/2 (T202/Y204), and P-S6 (S240/244). All images were taken at 200× magnification. Inset images of phospho-MET were amplified to show subcellular localization.

To determine the levels of MET and EGFR activation we performed immunostaining on four of the TNBC models (Figures 3 and Supplementary Table S1). Phospho-MET (Tyr1234/1235) was found to be strongest at the invasive edge of the tumors (Figures 3 and Supplementary Figure S1). This distinct pattern of increased MET activation near the invasive tumor front has been previously observed in non-small cell lung cancer and melanoma [35, 36]. We also observed unique phospho-MET (subsequently referred to as P-MET) expression patterns in each TNBC model. For example, PDX lines 109 and 124 had strong cytoplasmic and moderate nuclear P-MET expression, whereas P-MET was more predominant in the membrane in 200 and the nucleus in 201 (Figure 3, inset images). The phospho-MET antibody used in these studies is targeted to the cytoplasmic domain (near Y1234/Y1235). Therefore, it is possible that this nuclear signal is a cytoplasmic fragment of MET which has been observed by others [37]. Conversely, P-EGFR (Y1068) staining (using an antibody targeted to the cytoplasmic region near Y1068) was observed predominantly in the membrane of all the PDX lines. We also observed enhanced P-EGFR expression at the tumor periphery similar to P-MET.

Elevated MET and EGFR are able to activate numerous diverse signaling pathways that promote cell growth, invasion, angiogenesis, and cell survival [38, 39]. Two of the predominant signaling pathways activated by MET and EGFR are Ras/ERK and PI3K/AKT. The Ras/ERK and PI3K/AKT pathways are considered to be essential for tumor progression and both pathways are frequently associated with therapeutic resistance to TKIs [40]. To evaluate the baseline Ras/ERK signaling in our TNBC PDX models, we evaluated phospho-ERK1/2 (T202/Y204). ERK activation was significantly higher in PDX lines 109 and 124 compared to 200 and 201 (Figure 3). ERK is highly expressed in endothelial cells; therefore some of the staining observed can be attributed to the tumor vasculature, which is particularly evident in 201. ERK activation was also localized near the tumor periphery (Supplementary Figure S1) but this pattern was much less restricted than P-MET and P-EGFR. Activation of the PI3K pathway was confirmed using immunohistochemical analysis for the downstream target S6 ribosomal protein. Strong P-S6 staining was observed in all of the TNBC lines, however P-S6 activity was observed throughout the viable tumors. This pattern of S6 phosphorylation differed from P-ERK, P-EGFR, and P-MET which intensified near tumor periphery (Supplementary Figure S1). These results demonstrate that the ability of PDX models to recapitulate the RTK heterogeneity and downstream signaling that we observe in human TNBC tissue.

### Response to MET inhibition is associated with *MET* gene copy number gain

**Figure 4:**
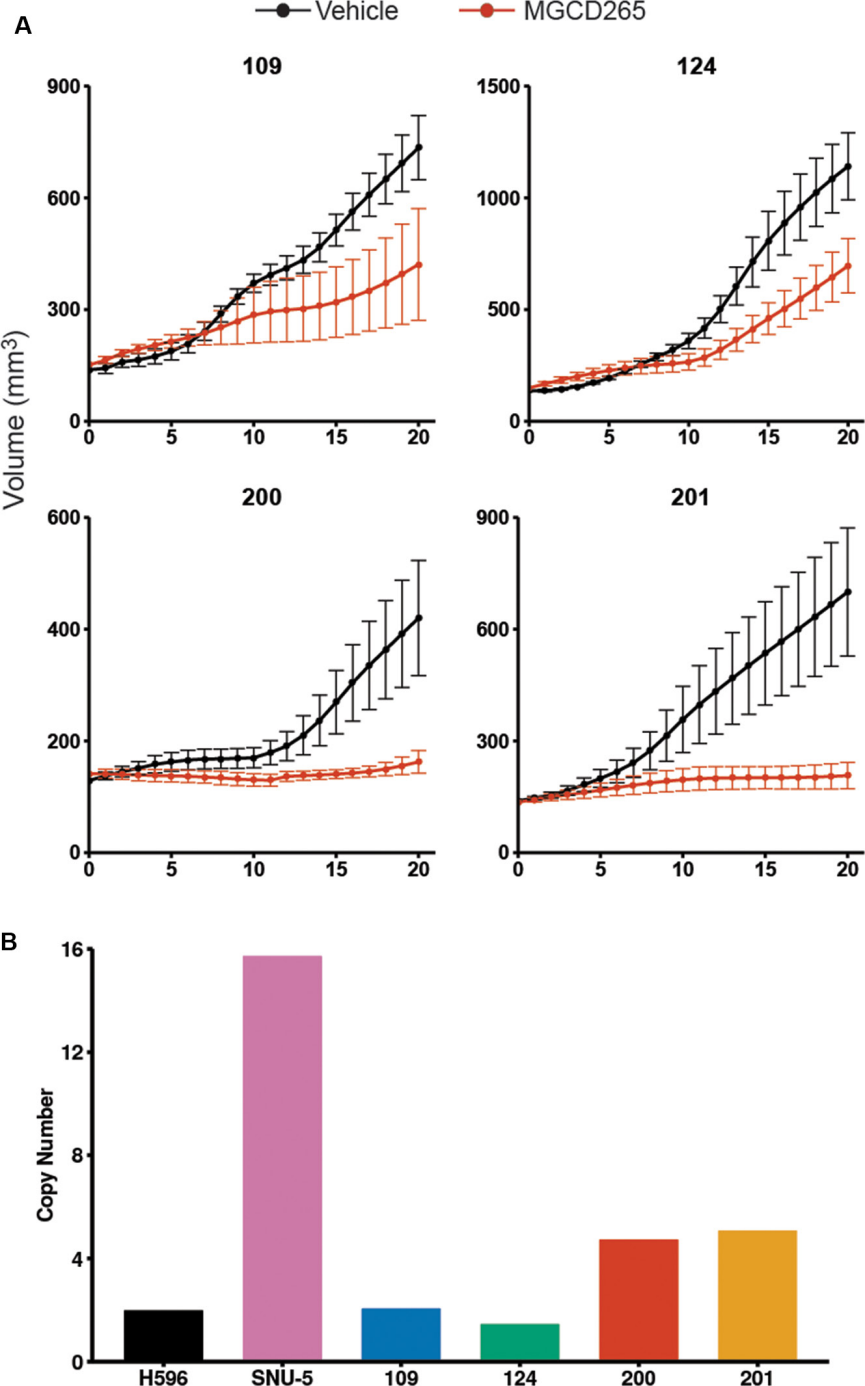
Monotherapy with MGCD265 inhibits TNBC tumor growth *in vivo* (**A**) Growth of TNBC PDX tumors were significantly inhibited by 40 mg/kg MGCD265 treatment (109 PDX, *p* < 0.05; 124, 200, and 201 *p* < 0.005). Linear mixed-effects modeling was used to test for significant differences in tumor growth. (**B**) A copy number variation assay was run on the TNBC lines to determine the *MET* copy number variation. H596 lung cells were used as a *MET* diploid control and SNU-5 gastric carcinoma cells which have high *MET* amplification were used as a positive control.

Since MET expression and its downstream signaling are strongly present in TNBC and our PDX models, we evaluated the effect of monotherapy with the MET inhibitor MGCD265. MGCD265 is an oral, multi-targeted, receptor tyrosine kinase inhibitor that targets MET, AXL, and PDGFR and is in clinical trials for non-small cell lung cancer (NSCLC), head and neck cancers, and advanced malignancies. Tumors were transplanted into NSG-SCID animals and when the tumors reached 150 mm^3^, animals were treated for 3 weeks. We observed significant tumor growth inhibition in all four TNBC PDX models (109 PDX, *p = 0.038;* 124, 200, and 201 PDX, *p = 0.001, 0.005, and 0.002* respectively) (95% CIs for estimated difference between MGCD265 and vehicle 109: [−35.817, −1.044], 124: [−0.06, −0.015] log transformed, 200: [−20.988, −3.657], 201: [−41.547, −9202]). Interestingly, treatment response positivity correlated *MET* gene copy number (Figure 4). For example, treatment response to MGCD265 was statistically significant in primary breast cancer tumorgrafts 109 and 124, yet there was a strong upward growth trend even after 3 weeks of treatment. Both 109 and 124 tumorgrafts have high MET expression (Figure 2) yet are not amplified at the *MET* locus (Figure 4B). In contrast, tumorgrafts 200 and 201 were derived from metastatic TNBCs, have 4–5 copies of *MET* (Figure 4B), and have a complete tumor growth inhibition to MET monotherapy with MGCD265 (Figure 4A). These results indicate that even though MET monotherapy is effective in high expressing TNBC tumorgrafts, TNBCs with *MET* gene copy number gain are more likely to have a complete response to MET inhibition possibly due to an ‘addiction’ to amplified *MET*. Therefore, combinations of targeted therapies may be necessary in TNBCs that do not involve *MET* gene copy number gains and coexpress alternate RTKs.

### Combined MET and EGFR inhibition eliminates variable treatment response in TNBC PDX models

**Figure 5:**
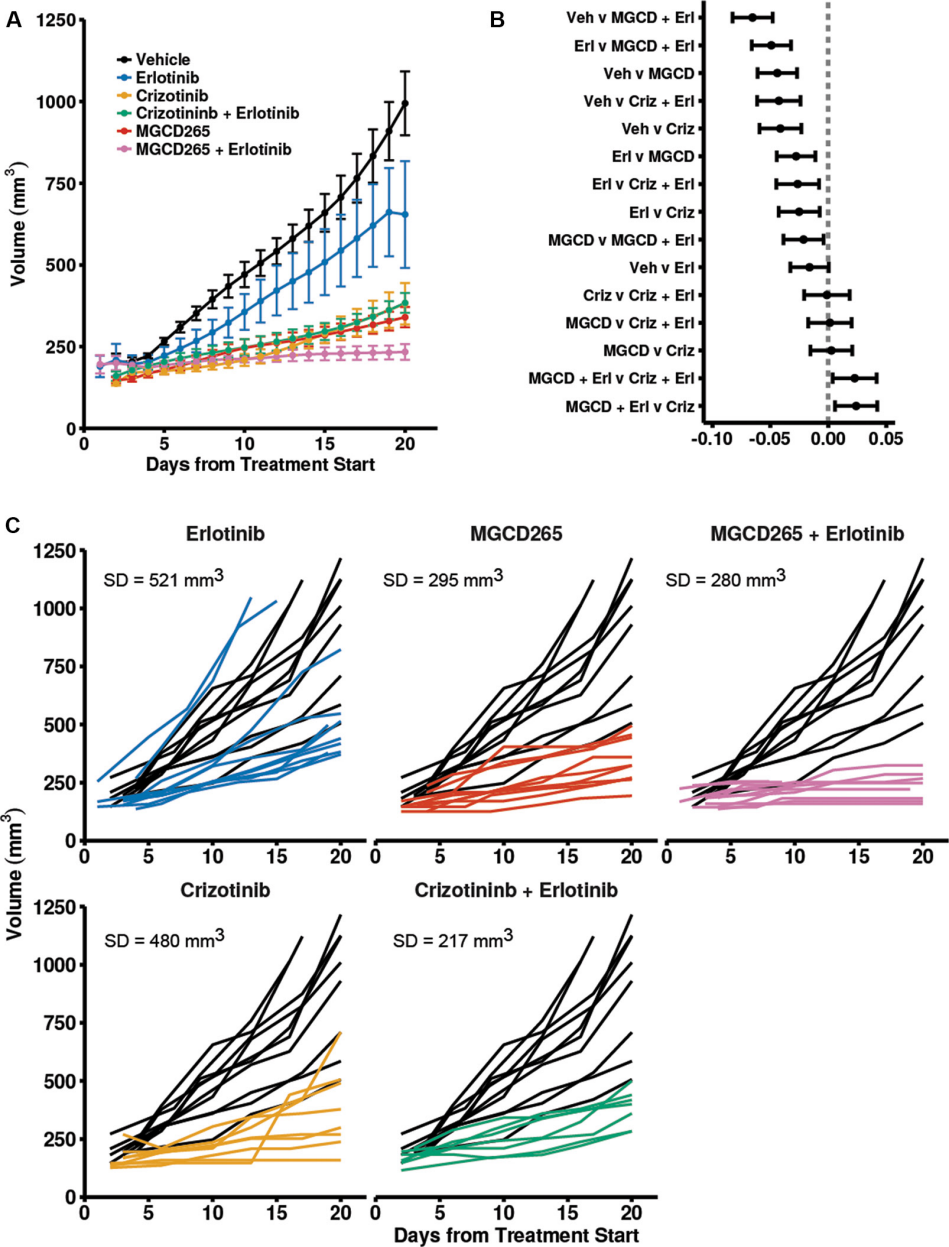
Combined MET and EGFR inhibition is more effective than monotherapy in TNBC PDX models (**A**) Growth of TNBC 109 PDX tumors were significantly inhibited by monotherapy of MGCD265 (40 mg/kg), erlotinib (50 mg/kg), or crizotinib (50 mg/kg) and combination therapy of MGCD265 plus erlotinib or crizotinib plus erlotinib. Plot displays LOESS interpolated mean tumor volume and SE. (**B**) Pairwise comparisons reveal that combined MET and EGFR inhibition is more effective than monotherapy. Plot shows 95% confidence intervals for each pairwise difference between the growth rates of the five treatments (tumor volumes were log transformed). If the interval is to the left of the vertical line, then the treatment on the right-hand side of the “v” had a significantly slower growth rate. (**C**) Individual growth curves of treatment compared to vehicle reveal an increased variability in response to monotherapy of a MET or EGFR TKI. Linear mixed-effects modeling was used test for significant differences in tumor growth. All multiple comparisons were adjusted for multiple testing using a false discovery rate correction.

Because of the partial responses to MET monotherapy observed (Figure 4A) and previous studies indicating MET-EGFR crosstalk in therapeutic resistance, we evaluated combined MET and EGFR inhibition in a primary TNBC tumorgraft (109) with the MET inhibitors MGCD265 or crizotinib (Xalkori) and the EGFR inhibitor erlotinib (Tarceva). We observed a significant response to monotherapy with MET or EGFR inhibitors in the 109 TNBC line; tumors in the crizotinib treated mice grew an average of 0.044 log (mm^3^) per day less than the vehicle, 0.016 less in the erlotinib treated mice, and 0.061 less in the MGCD265 treated mice (crizotinib: *p < 0.001, 95*% CI [−0.059, −0.027]; erlotinib *p* = 0.052, 95% CI [−0.033, 0.000], MGCD265: *p* < 0.001, 95% CI [−0.061, −0.027]). In the 109 TNBC tumors, MET inhibition with MGCD265 or crizotinib was significantly more effective than erlotinib treatment alone (*p* = 0.001 and 0.005; 95% CI [−0.044, −0.011] and [−0.043, −0.008] respectively)) (Figure 5A–5B). Overall, combined treatment of MGCD265 plus erlotinib was the most effective treatment in the 109 TNBC line (*p* = 0.017, 95% CI difference between the combo therapies [−0.042, −0.004]). If we examine the individual growth curves for each tumor in the treatment compared to vehicle control groups, we can assess the variability in response to each treatment. For this analysis we used the last time point with a high number of observations (> 3 tumors) to evaluate the standard deviation in response. In the 109 TNBC tumors, erlotinib or MGCD265 treatment alone was significantly effective at inhibiting tumor growth (Figure 5A); however the variation in response was 521 mm^3^ with erlotinib treatment compared to 295 mm^3^ with MGCD265 treatment (Figure 5C). The variation decreases to 280 mm^3^ when a combined treatment of MGCD265 + erlotinib is given. A similar decrease in treatment variability is seen with crizotinib + erlotinib (217 mm^3^). To ensure this response to combined MET and EGFR inhibition was not limited to the 109 TNBC model, we examine the efficacy of MGCD265 and/or erlotinib treatment in the 124 TNBC tumorgrafts (Supplementary Figure S2). Here we show a similar result in which MGCD265 plus erlotinib was the most effective treatment strategy. Even though erlotinib or MGCD265 monotherapy mediated tumor growth inhibition were statistically significant, the growth curves of tumors in each monotherapy group had an upward growth trend at the end of the study. These results underscore the potential for combined RTK inhibition to eliminate treatment variability and resistance potential in patients.

**Figure 6:**
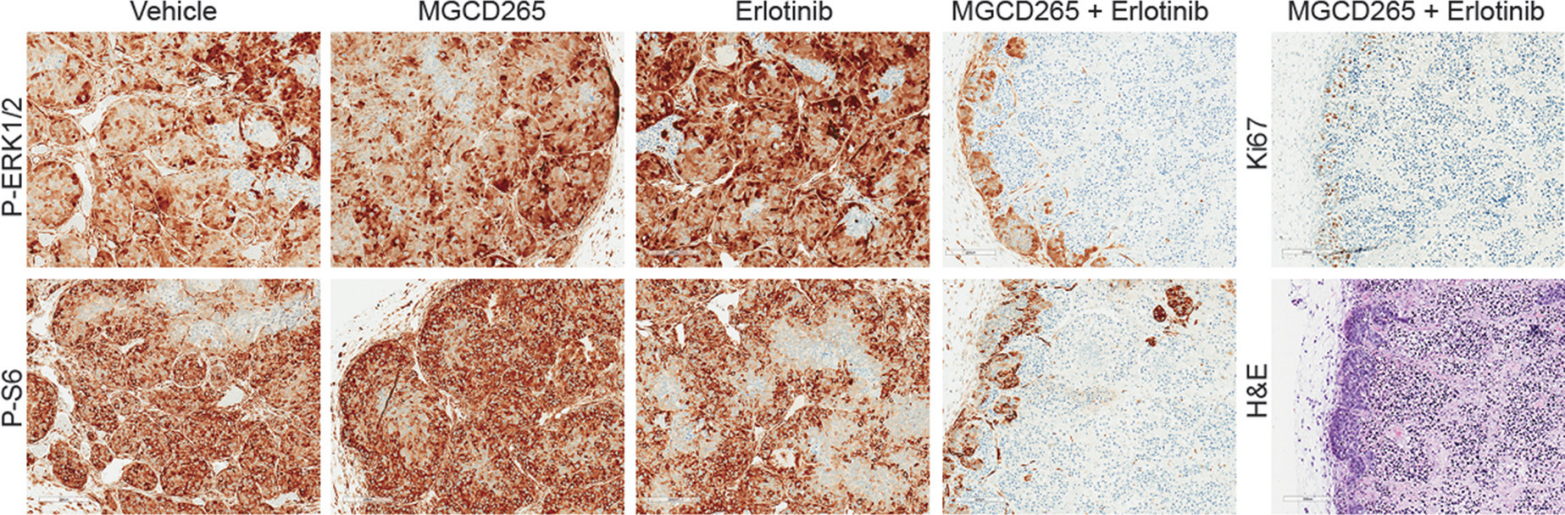
Decreased ERK and AKT/mTOR signaling only observed with combined MET and EGFR inhibition Downstream MET and EGFR signaling was evaluated by immunostaining of pERK1/2 (T202/Y204) and pS6 (S240/244) in 124 tumorgrafts treated with vehicle, MGCD265, erlotinib, or MGCD265 + erlotinib for 3 days. Ki67 and H&E images for the dual MGCD265 + erlotinib treatment are shown in the right panel. All images were taken at 100× magnification.

To understand the effect of combined MET and EGFR treatment versus monotherapy on downstream signaling, we assessed ERK and AKT/mTOR activity in the 109 and 124 tumorgraft lines (Figure 6). Elevated levels of P-ERK and P-S6 were observed throughout the vehicle-treated tumors and were still present after 3 days of MGCD265 or erlotinib treatment. In contrast, a significant decrease in P-ERK and P-S6 levels were observed in tumors treated for 3 days with both MGCD265 and erlotinib. Ki67 and H&E staining reveal the significant decrease in proliferation and increase in necrosis in tumors treated with the MGCD265 and erlotinib combination (Figure 6, right panel). These findings support our tumor growth results shown in Figure 5 and indicate the efficacy of combined MET and EGFR inhibition on deterring crosstalk signaling through the ERK and AKT pathways. Overall, these results demonstrate the substantial efficacy of combined MET and EGFR inhibition in abrogating downstream RTK signaling, reducing tumor progression, and reducing tumor response variability.

## DISCUSSION

The fact that distant recurrence occurs in 34% of TNBC patients within 2 to 5 years from diagnosis underscores the urgent need for effective therapeutic strategies in both primary and metastatic TNBC [2]. The MET and EGFR tyrosine kinases are actionable therapeutic targets due to their high expression in TNBC and availability of several selective kinase inhibitors that are either FDA approved for other cancers or are in clinical trials. The success of trastuzumab in HER2^+^ breast cancer and EGFR inhibition with gefitinib or erlotinib in NSCLC demonstrate the promise of TKI treatment. Nevertheless, these responses are often not durable and acquired resistance to kinase inhibition drives tumor progression. These failures reveal our limited understanding of the plasticity of the kinome and the compensatory signaling mechanisms that cells use to support survival during targeted kinase inhibition. For example in NSCLC, *MET* amplification drives resistance to the EGFR inhibitor gefitinib; whereas in ALK-positive NSCLC, ALK inhibition can be overcome by EGFR or KIT activation [23, 41]. These studies and others in glioblastoma, melanoma, colon, and several other cancers indicate that it is essential that we understand the landscape of RTK expression in order to predict effective therapeutic strategies and prevent acquired resistance.

Two critical signaling pathways that are maintained by RTK crosstalk and activation are the Ras/ERK and PI3K/AKT pathway. Several recent studies have revealed how coexpression of RTKs can promote signaling crosstalk and maintain active Ras/ERK and PI3K/AKT signaling in the presence of kinase inhibitors to ensure cell survival. To understand how ligand expression can promote resistance to kinase inhibitors, Wilson et al. performed a matrix analysis of six different RTK ligands in 41 cancer cell lines [7]. This study demonstrated that RTKs are frequently coexpressed in cancer cell lines even in “kinase-addicted” cells, such as those with *HER2* amplification or BRAF mutation. Moreover, drug sensitivity could be overcome in “kinase-addicted” cancer cells by expression of one or more RTK ligands. For example, HGF expression was able to overcome lapatinib-sensitivity in several HER2-dependent breast cancer cell lines. These findings support our previous study in HER2+ breast cancers in which we observed that the majority of HER2+ breast cancers coexpress MET and HER2 and a subset of the cells within these tumors are MET+/HER2+ [42]. We also demonstrated that increased activation of MET or HER2 compensated for loss of HER2 or MET respectively. Overall these studies indicate the “kinase-addicted” cancers still have significant signaling redundancy and the potential to become resistant to kinase inhibitors through the coexpression of other RTKs or their ligands.

Network modeling approaches have also identified specific groups of RTKs that are functionally redundant and compensate to maintain RTK signaling network activation. Using an RNAi perturbation strategy in isogenic cell lines, Wagner et al. evaluated the common and unique features of RTK signaling networks. Their results revealed that specific groups of RTKs exhibited functional redundancy by their ability to activate similar downstream signaling networks [5]. By evaluating the conserved sets of signaling pathways, RTKs can be grouped into three distinct classes: (i) an EGFR/FGFR1/MET class constituting EGFR, fibroblast growth factor receptor 1, and MET; (ii) an IGF-1R/NTRK2 class constituting insulin-like growth factor 1 receptor and neurotrophic tyrosine receptor kinase 2; and (iii) a PDGFRβ class constituting platelet-derived growth factor receptor β. In agreement with the findings of Wilson et al., this network analysis determined that RTK coexpression frequently correlated with resistance to a drug targeting another RTK of the same class. For example, EGFR, MET, and FGFR1 were highly expressed in 196 cell lines of carcinoma, melanoma, and glioma origin and high expression of EGFR correlated with decreased sensitivity to the MET inhibitor PHA665752. These findings were substantiated in a high-throughput secretome screen in which EGF and FGF were able to rescue MET inhibition in MET-dependent gastric carcinoma cells [43]. Conversely, HGF was able to rescue FGFR3 inhibition in bladder cancer cells. Importantly, in each of these cases, ligand mediated rescue could be prevented by combination treatment with RTK inhibitors. These studies indicate the significance of MET/EGFR coexpression in cancer and the potential efficacy of rational combination therapy.

In this study, we demonstrate the enhanced efficacy of targeting both MET and EGFR in TNBC. We used both primary and metastatic PDX TNBC models that have variable levels of MET and EGFR expression. Not only do these PDX models recapitulate the complex signaling networks present in TNBC, they also maintain the histological and molecular features present in human TNBC [30]. The fact that PDX models retain the characteristics of the primary tumor with high fidelity is an advantage over human cell line xenografts which only partially recapitulate breast tumor biology, metastatic progression, and response to therapy [44]. Using these preclinical TNBC models, we evaluated the efficacy of monotherapy with the MET inhibitor MGCD265. Even though MGCD265 treatment significantly impaired tumor growth in all of the TNBC lines, complete growth inhibition after 3 weeks of treatment was only observed in the TNBC lines that had 4–5 *MET* copies and a positive growth trend can be observed in tumors without *MET* gene copy gain (Figure 4A). These results agree with studies showing that TKI inhibition is often more effective in tumors harboring genomic amplified kinases, such as *HER2*-amplified breast cancers and *MET*-amplified lung cancers [45, 46]. Even though amplified RTKs can be exquisitely sensitive to kinase inhibition, recent studies in esophagogastric carcinomas have demonstrated that 40–50% of *MET*-amplified tumors coharbor amplified *HER2* or *EGFR* [47]. The coexpression of MET with HER2 or EGFR maintains redundant kinome signaling by which ERK and AKT signaling activity is sustained. Here we demonstrate that combined MET and EGFR inhibition with MGCD265+erlotinib or crizotinib+erlotinib were the most effective treatments and significantly decreased the variability in treatment response compared to monotherapy. Overall these results present a therapeutic treatment strategy that may be effective in a high percentage of TNBC patients. Furthermore, these studies support the findings of targeting multiple RTKs to prevent signaling crosstalk and resistance to kinase inhibitors.

## MATERIALS AND METHODS

### Primary human breast tumors

Primary breast cancer specimens were collected at Spectrum Health from 2010–2015 under a protocol approved by the Institutional Review Boards of both Spectrum Health and the Van Andel Research Institute. Histopathology and ER, PR, and ERBB2 status were determined by a clinical pathologist at Spectrum Health.

### Development of patient-derived tumorgrafts and *in vivo* testing of TKIs

Tumor tissue was obtained during standard surgical procedures and fresh clinical breast cancer tissue was placed into the 4th mammary fat pad of NSG-SCID female mice via trocar. Bulk tumor pieces (1–3 mm) were placed into the mammary fat pad using a 10 g trochar. For tumors that successfully transplanted, these were serially passaged into NSG-SCID and/or athymic nude female mice for tumor studies. For testing of efficacy of TKIs, bulk tumor pieces were transplanted and tumor growth was evaluated twice weekly. When tumor volume reached approximately 150 mm^3^, the mice were randomized into the following treatment groups: 1) vehicle; 2) MGCD265 [40 mg/kg], 3) erlotinib [75 mg/kg, QD], 4) crizotinib [50 mg/kg, BID], 5) MGCD + erlotinib, or 6) crizotinib + erlotinib. MGCD265 was obtained from Mirati Therapeutics. Crizotinib and erlotinib were purchased from LC Labs. The tumors were measured twice weekly using a caliper and the tumor volumes (length × width × depth) were calculated. Animal studies were approved by the Institutional Animal Care and Use Committee (IACUC) of the Van Andel Research Institute (VARI).

### Immunohistochemical staining and analysis

For immunohistochemical staining, heat-induced epitope retrieval with an EDTA/borate/Tris buffer (Ventana Medical Systems) was used and detection was performed with a Ventana Discovery XT or Ventana Discovery Ultra immunostainer (Ventana Medical Systems). Primary antibodies were revealed using an UltraMap anti-Rabbit DAB (brown) detection kit (Ventana Medical Systems); hematoxylin was employed as a nuclear counterstain. Primary antibodies for staining were Met-4 [48], EGFR (DAK-H1-WT, Dako), and the following antibodies from Cell Signaling Technology (Beverly, Massachusetts): P-MET (Y1234/Y1235; D26), P-EGFR (Y1068; D7A5), P-MAPK (Thr202/Tyr204; #9101), and P-S6 (Ser240/244).

### Copy number variation assay

MET gene copy number was determined using genomic DNA from purified organoids (PureLink Genomic DNA mini kit; Life Technologies) according to manufacturer's instructions. H596 lung adenocarcinoma cells which have two known copies of MET and SNU-5 gastric carcinoma cells with known *MET* amplification were used as controls. For each sample, 20 ng gDNA was used in a duplex, real-time PCR reaction with a set of commercially available TaqMan Copy Number Assay primers specific for MET (Hs04945184_cn or Hs04959714_cn; Life Technologies) along with the TaqMan Copy Number Reference Assay, RNase P, in a 96-well reaction plate. PCR plates were run on the StepOnePlus Real-Time PCR machine (Applied Biosystems) and copy number was determined using CopyCaller Software (Life Technologies).

### Statistical analysis

Linear mixed-effects models, with the appropriate linear contrasts were used to test for significant differences in drug response across treatment arms. A false discovery rate correction was used to adjust tests for multiple testing. If a given PDX model had residuals that greatly deviated from normality (determined visually via QQ-plots), tumor was natural log transformed. All outliers were examined for potential experimental issues.

## SUPPLEMENTARY MATERIALS


