# Supplementary material for: Targeting MET and EGFR crosstalk signaling in triple-negative breast cancers
Source: Oncotarget. 2016 Sep 16;7(43):69903–15. doi: 10.18632/oncotarget.12065 (PMC5342523; doi:10.18632/oncotarget.12065)
Supplement: Supplementary file 1 [file oncotarget-07-69903-s001.pdf]

Targeting MET and EGFR crosstalk signaling in triple-negative breast cancers

Supplementary Materials

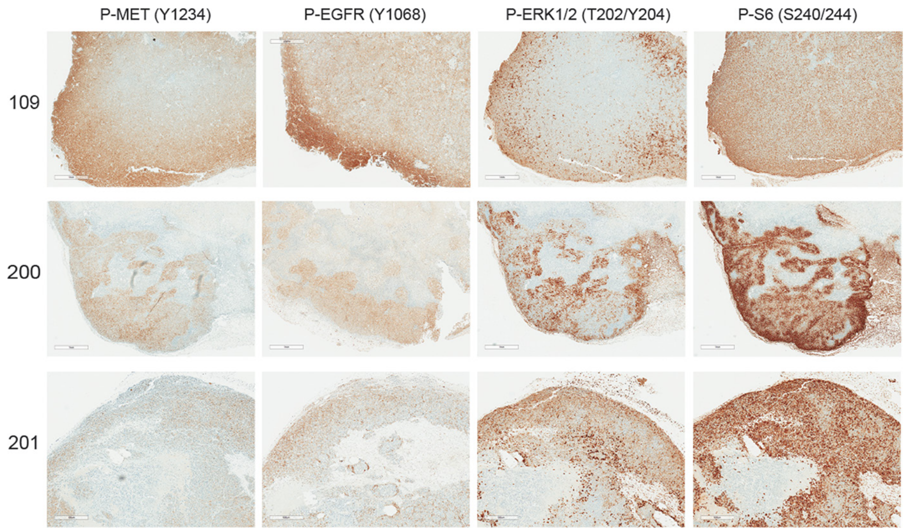

Supplementary Figure S1: MET and EGFR signaling is highly activated in TNBC. MET, EGFR, and ERK activation is observed near the invasive edge of the tumors, whereas P-S6 is observed throughout the tumors. MET and EGFR activation was determined by immunostaining of P-MET (Tyr1234/1235), P-EGFR (Y1068), P-ERK1/2 (T202/Y204), and P-S6 (S240/244). All images were taken at 20× magnification.

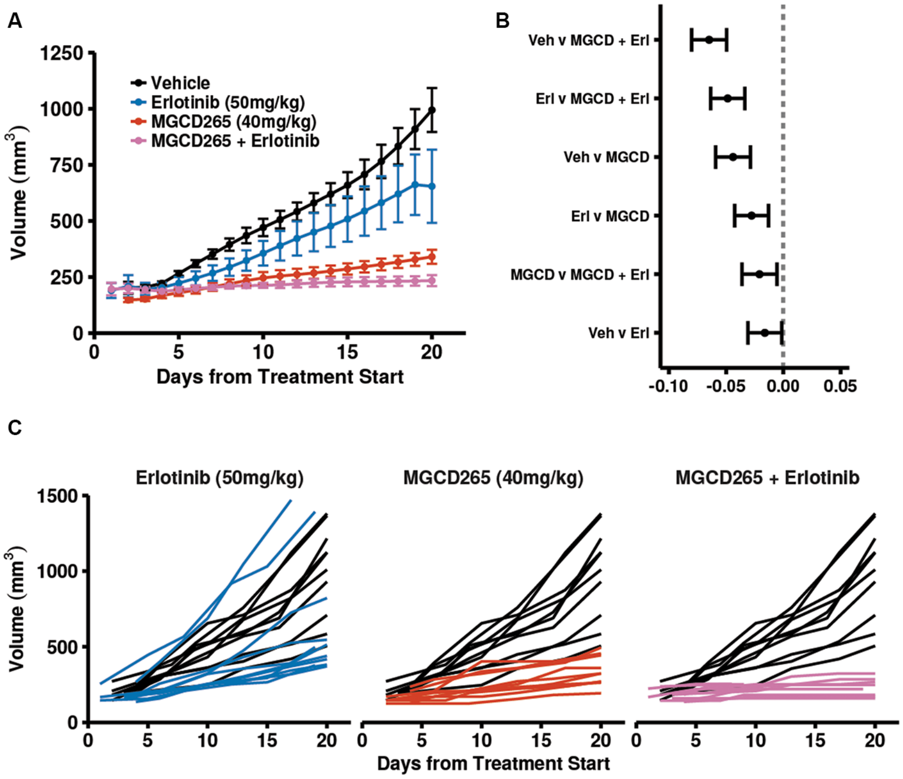

Supplementary Figure S2: Combined MET and EGFR inhibition is more effective than monotherapy in TNBC 124 tumorgrafts. (**A**) Growth of TNBC 124 PDX tumors were significantly inhibited by monotherapy of MGCD265 (40 mg/kg; $p < 0.001$), erlotinib (50 mg/kg; $p < 0.01$), and combination therapy of MGCD265 + erlotinib ($p < 0.00001$). Plot displays LOESS interpolated mean tumor volume and SE. (**B**) Pairwise comparisons reveal that combined MET and EGFR inhibition is more effective than monotherapy. Plot shows 95% confidence intervals for each pairwise difference between the growth rates of the three treatments (tumor volumes were log transformed). If the interval is to the left of the vertical line, then the treatment on the right-hand side of the "v" had a significantly slower growth rate. (**C**) Individual growth curves of treatment compared to vehicle demonstrate the variability in response to erlotinib (SD = 225 mm^3), MGCD265 (SD = 171 mm^3), or MGCD265 + erlotinib (SD = 86 mm^3). Linear mixed-effects modeling was used test for significant differences in tumor growth. All multiple comparisons were adjusted for multiple testing using a false discovery rate correction.

Supplementary Table S1: Summary of MET and EGFR expression and activity in TNBC PDX lines

PDX	MET	EGFR	P-MET	P-EGFR
109	+++	+++	+++	+++
113	++	++	ND	ND
124	+++	++	+++	+++
200	+++	+++	++	++
201	++	++	++	++

Relative expression of MET, EGFR, P-MET, and P-EGFR expression for each PDX model is shown. Scoring range was from 0 to +++ are were based on immunohistochemical stains as represented in Figures 2 and 3. MET localization varied between the membrane and cytoplasm. We observed stronger cytoplasmic staining in PDX lines 113 and 201 compared to PDX lines 109 and 124 which had intense membrane MET staining.cytoplasmic staining in PDX lines 113 and 201 compared to PDX lines 109 and 124 which had intense membrane MET staining.
